# Supplementary figures and images for: The S-palmitoylome and DHHC-PAT interactome of Drosophila melanogaster S2R+ cells indicate a high degree of conservation to mammalian palmitoylomes
Source: PLoS One. 2022 Aug 12;17(8):e0261543. doi: 10.1371/journal.pone.0261543 (PMC9374236; doi:10.1371/journal.pone.0261543)

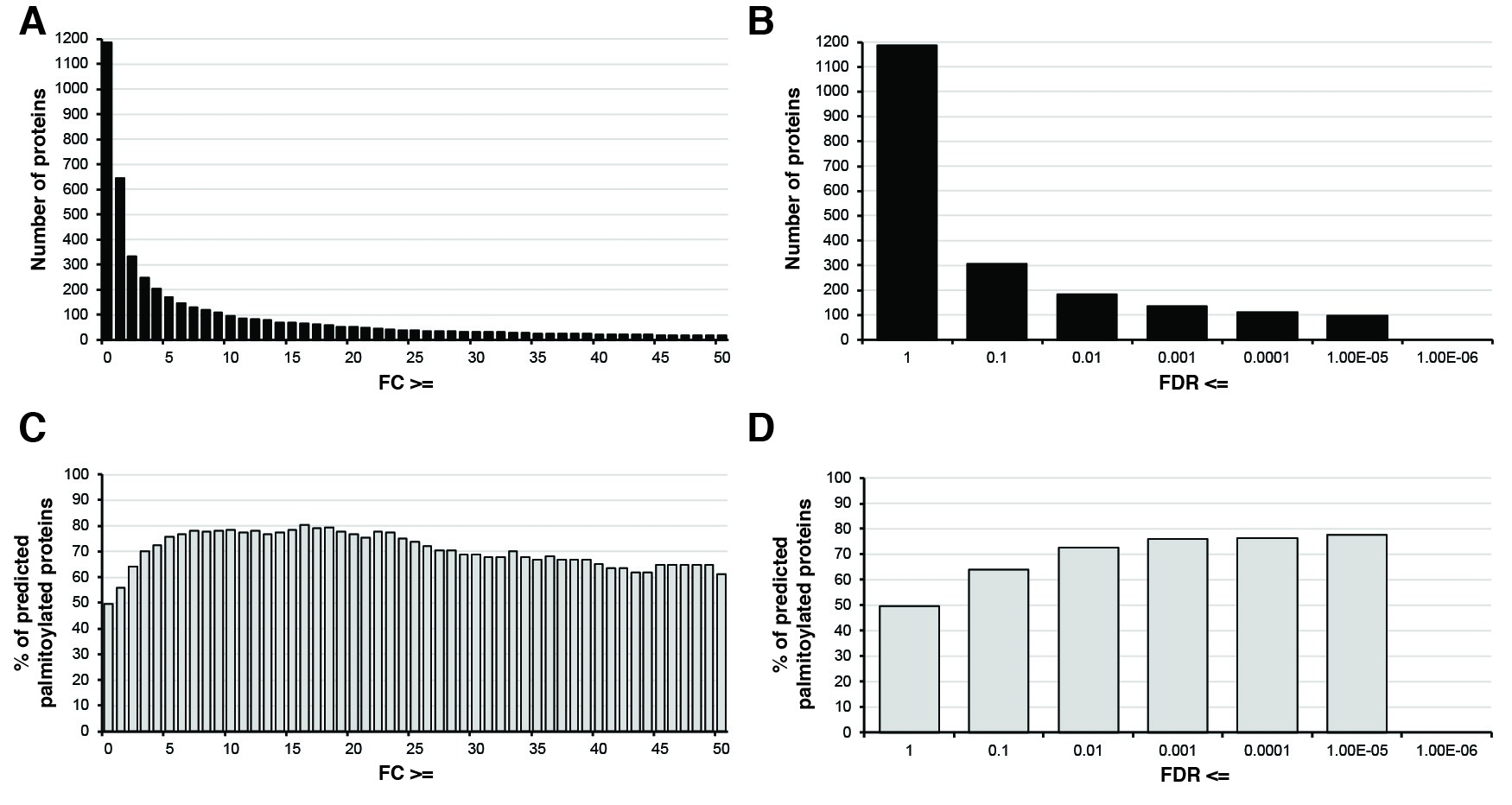

Supplement: S1 Fig — Variation of the total number of proteins in the Acyl-RAC dataset (1188 proteins in total) and the fraction of those predicted to be palmitoylated by CSS-Palm with increasing FC and FDR cut-offs. (A) Bars represent the total number of proteins with an FC > = the value indicated in the x axis. (B) Bars represent the total number of proteins with an FDR < = the value indicated in the x axis. In (C) and (D) bars represent the fraction of proteins that are predicted as palmitoylated regarding the totals indicated in the barplots above. (TIF) [file pone.0261543.s001.tif]

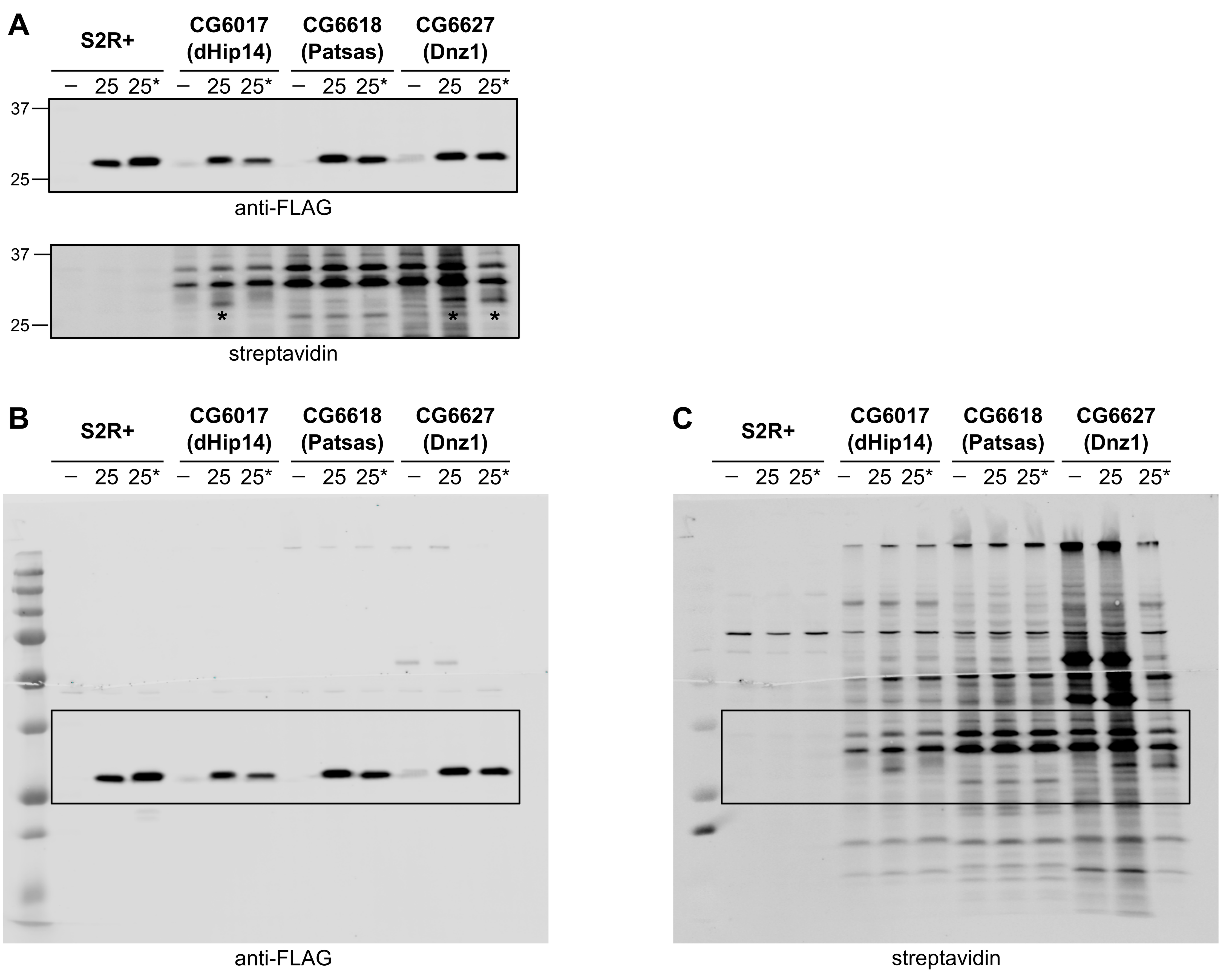

Supplement: S2 Fig — (A). Full blots with rectangles indicating the sections used for panel (A) are shown for the anti-FLAG antibody (B) and streptavidin (C). (TIF) [file pone.0261543.s002.tif]

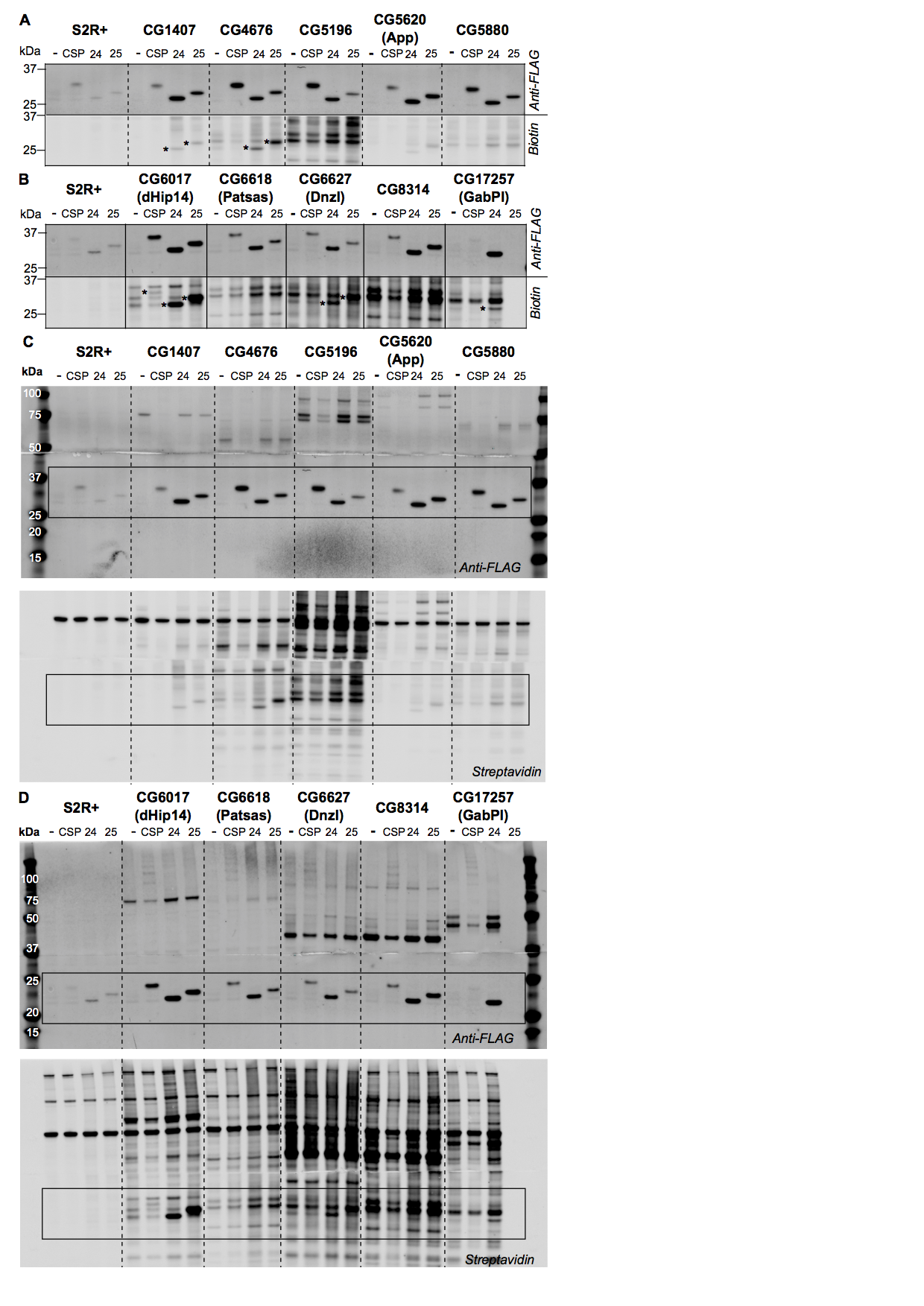

Supplement: S3 Fig — (A, B) Representative blots are shown. FLAG-dSNAP24/25/CSP were detected using an anti-FLAG primary antibody (upper panel) and biotin was detected using a Streptavidin probe (lower panel). (C) original blot relative to panel A. (D) original blot relative to panel B. (C, D) Boxed areas indicate the cropped region in the corresponding panels. Biotinylation and FLAG-tagged proteins were detected on the same blot membrane using two different fluorophores on a Li-Cor Odyssey. (TIF) [file pone.0261543.s003.tif]

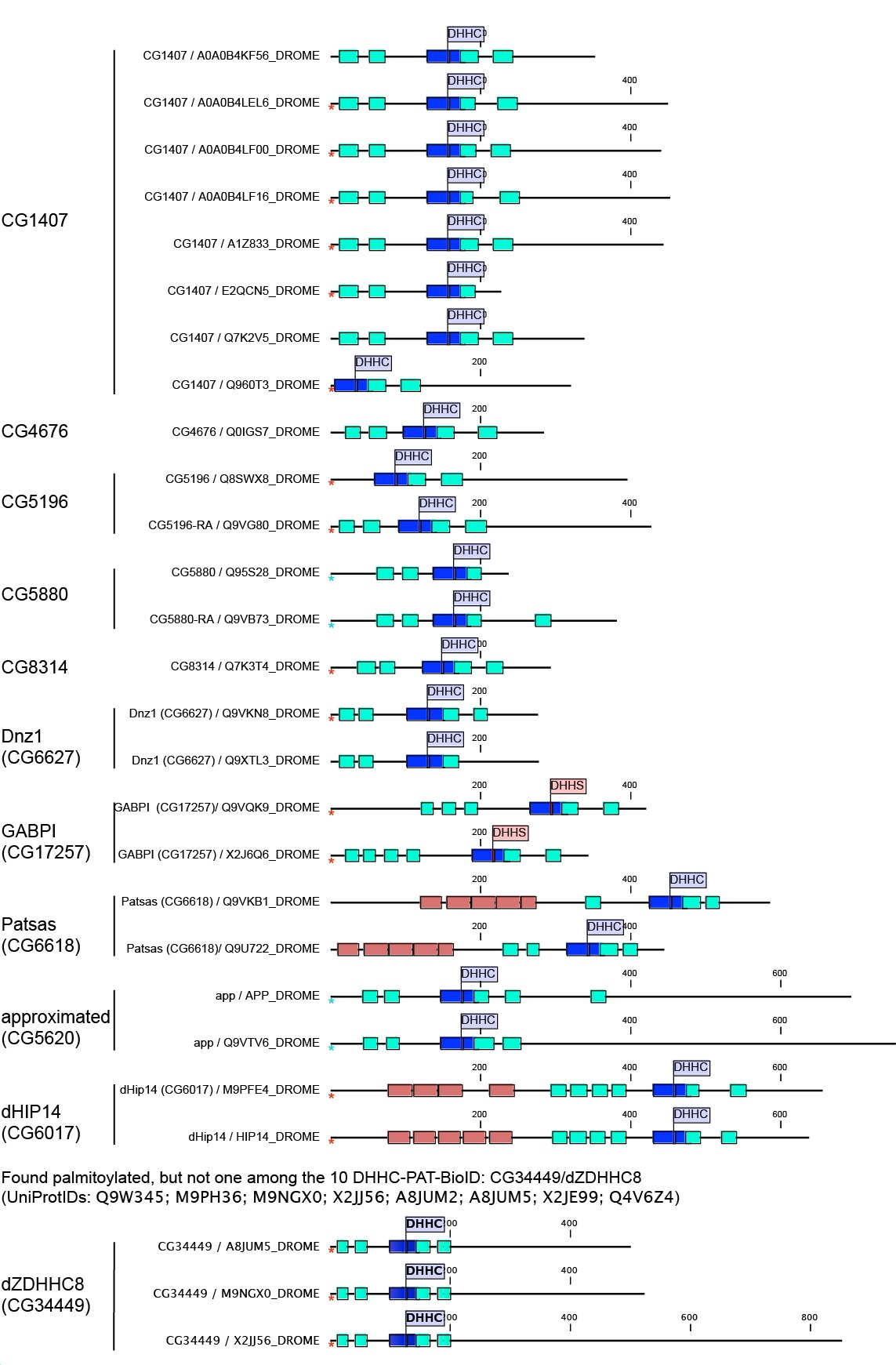

Supplement: S4 Fig — Amino acid sequences were obtained from UniProt.org, UniProt IDs are shown; cyan boxes = trans membrane domains; blue boxes = cystein rich domain (CRD) with DHHC/DHHS site indicated by a flag; red boxes = ankyrin repeats; N-termini are on the left hand side; except for dZDHHC8 (CG34448) all DHHC-PATs are shown at the same scale. Asterisks indicate DHHC-PATs recovered by acyl-RAC, red asterisks = normal/high confidence group, green asterisks = below threshold for palmitoylation but identified by mass spectrometry. (TIF) [file pone.0261543.s004.tif]

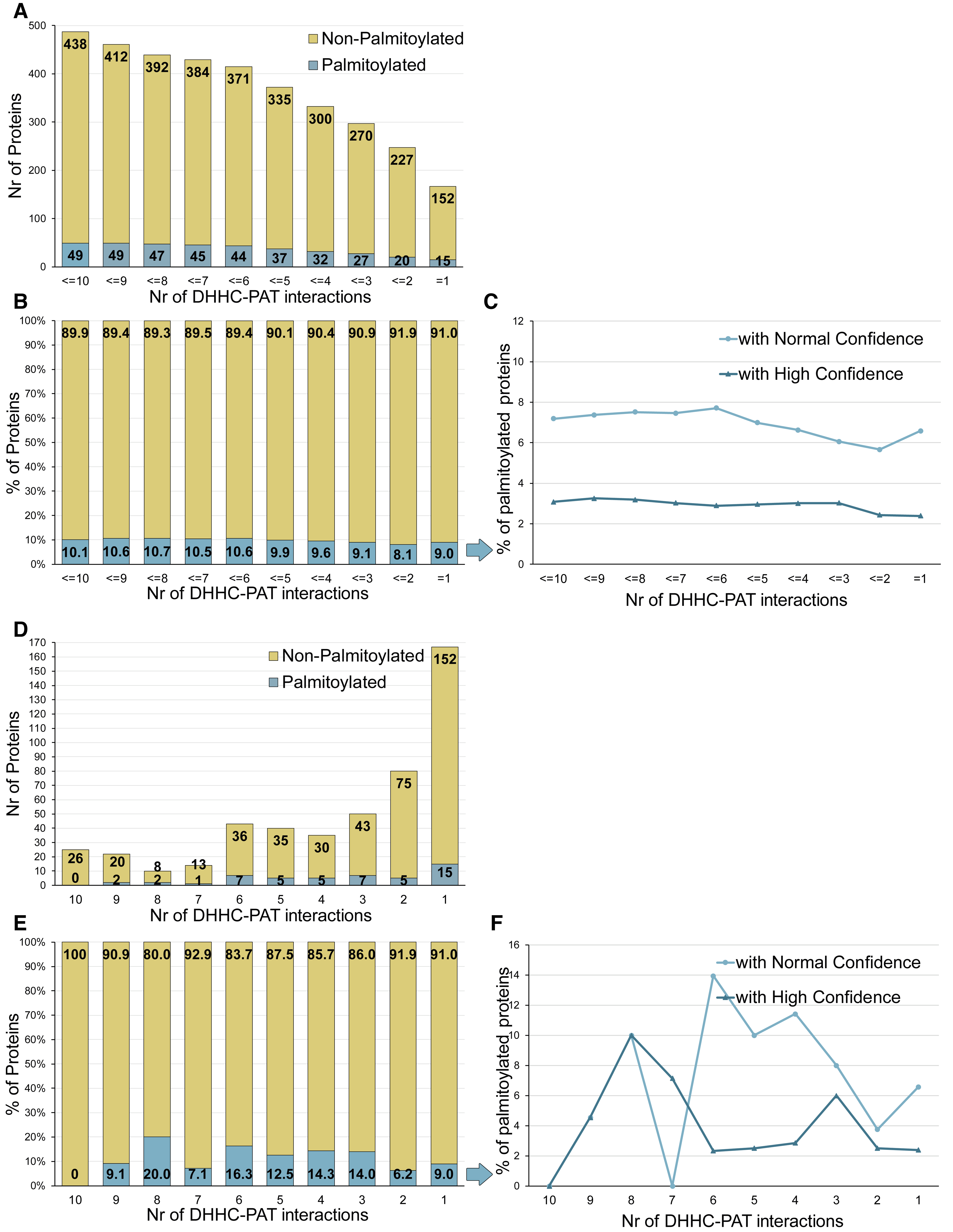

Supplement: S5 Fig — Absolute and relative number of proteins (y-axis) that are interacting with a given number of DHHC-PATs (y-axis; Nr of DHHC-PAT interactions). The number of DHHC-PAT interactions as a threshold (A-C) or fixed number of interations (D-F). Number of proteins as absolute numbers (A and D) or as (B, C, D, and E). Comparison of percentages of palmitoylated proteins that were determined with ‘normal confidence’ or with ‘high confidence’ (C and D). (TIF) [file pone.0261543.s005.tif]

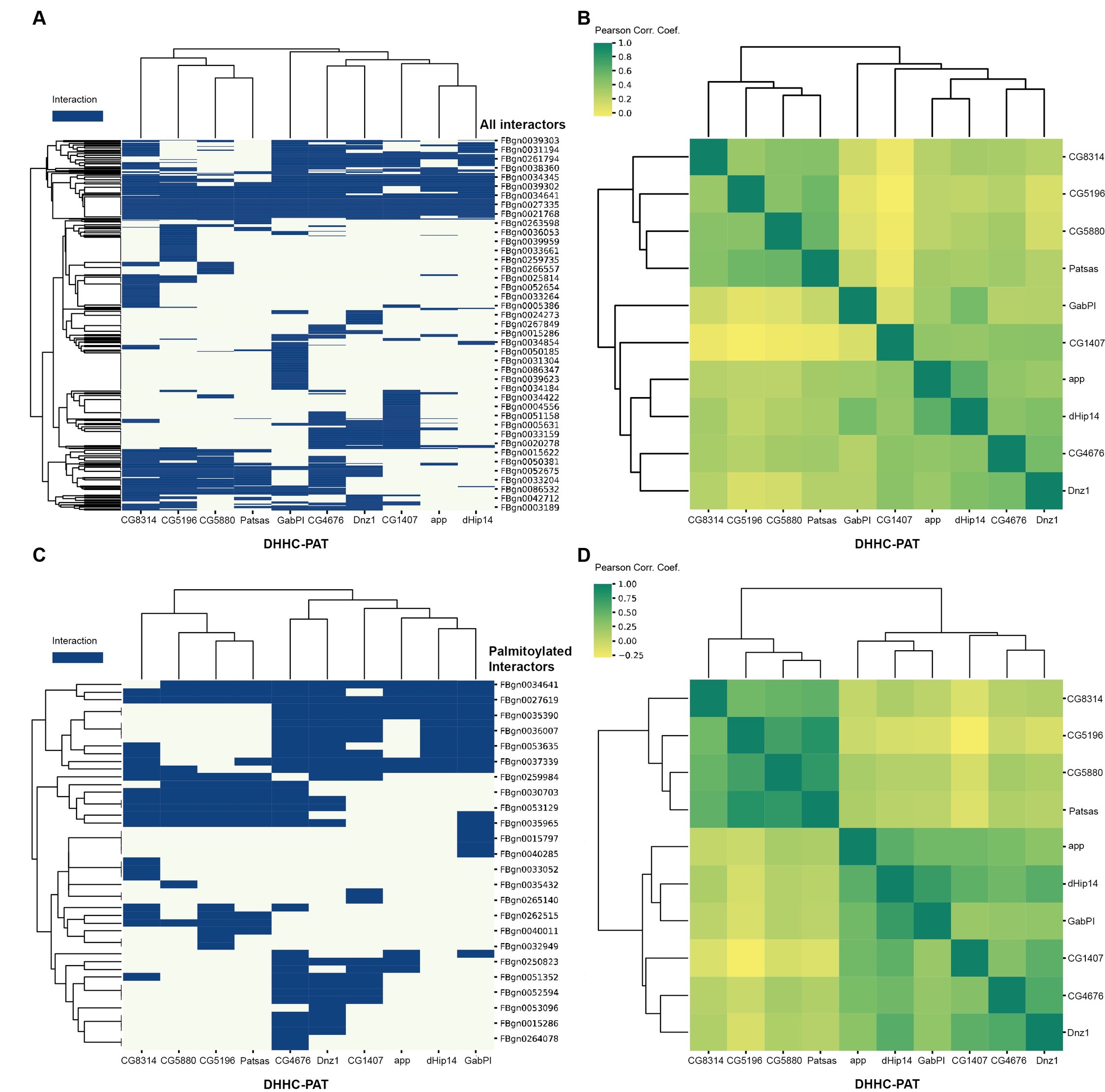

Supplement: S6 Fig — (A) Hierarchical cluster map of DHHC-PATs (x axis) on the basis of whether they interact (blue cells) or not (white cells) with each putative interactor (proteins that at least interact with one DHHC-PAT). (B) Hierarchical clustering of the Pearson correlation coefficients between each DHHC-PAT pair determined from their interactors spectra. (C-D) Same two plots but determined considering only interactions with proteins that were experimentally defined to be S-palmitoylated. Two broad groups are consistently defined in all plots, although the hierarchy within them is not equally consistent. The first group comprises enzymes CG8314, CG5196, CG5880 and Patsas, and the second one, GabPi, app, Hip14, Dnz1, CG1407, CG4676. Plots were made using the clustermap function of the Seaborn library for Python. (TIF) [file pone.0261543.s006.tif]

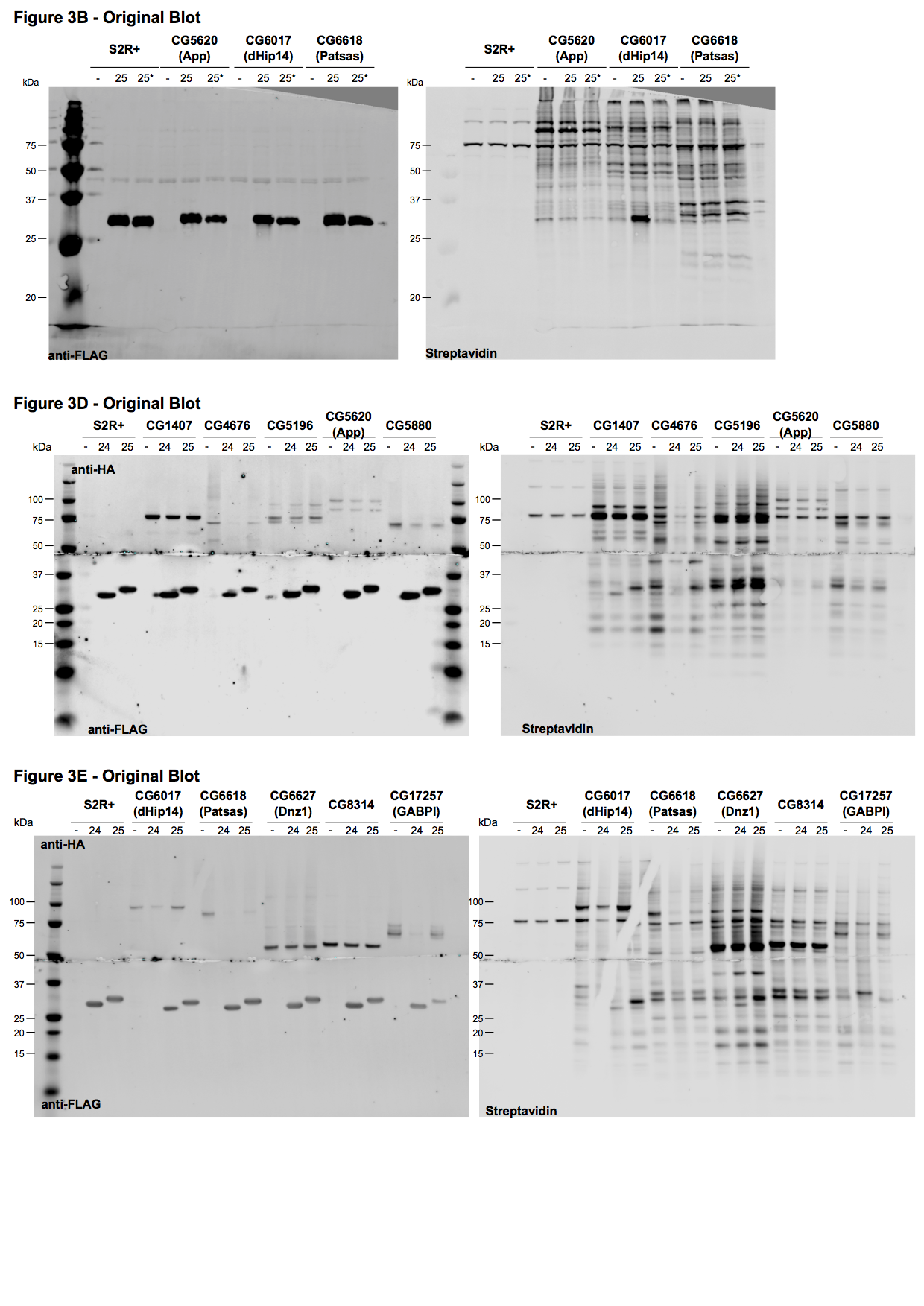

Supplement: S1 Raw image — (TIF) [file pone.0261543.s007.tif]
