# Supplementary material for: The S-palmitoylome and DHHC-PAT interactome of Drosophila melanogaster S2R+ cells indicate a high degree of conservation to mammalian palmitoylomes
Source: PLoS One. 2022 Aug 12;17(8):e0261543. doi: 10.1371/journal.pone.0261543 (PMC9374236; doi:10.1371/journal.pone.0261543)
Supplement: S1 Table — (DOCX) [file pone.0261543.s008.docx]

## **S1** **Table.** **Primers for molecular cloning.**

| primer name | 5´ -sequence- 3´ |
| --- | --- |
| FLAG-dSnap24 |  |
| NotI-FLAG-dSnap24 | aaaaGCGGCCGCaccATGgactacaaagacgatgacgacaag-ATGGCCGCCGTGGAGAAT |
| XhoI-dSnap24-R | AAAActcgagGTTTCGGGACGTGGCCGTT |
| FLAG-dSnap25 |  |
| NotI-FLAG-dSnap25 | aaaaGCGGCCGCaccATGgactacaaagacgatgacgacaag-ATGCCAGCGGATCCATCTG |
| XhoI-dSnap25-R | AAAActcgagGTCGCCCTGCAATGTTTTTACTT |
| FLAG-dCSP |  |
| NotI-FLAG-dCSP | aaaaGCGGCCGCaccATGgactacaaagacgatgacgacaag-ATGAGCGCACCTGGCATG |
| XhoI-dCSP-R1 | AAAActcgagTAGGGGCTTGTGTTTTCTGTGTCTG |
| XhoI-dCSP-R2 | AAAActcgagAAGCGACGACACAAATGCCACAAA |
| mycBioID and mycBioID2 | |
| NotI-Myc-F | aaaaGCGGCCGCaccatggaacaaaaactcatctc |
| XhoI-BirA-R | AAAActcgaggcttctctgcgcttctca |
| XhoI-BioID2-R | AAAActcgagGCTTCTTCTCAGGCTGAACTC |
| 5´UTR-Hip14-BioID-3´UTR - Enhanced Gibson Assembly | |
| dHip14 5´ UTR F | ggccgcggctcgagggtaccaggccttgatca-CCACACATTCAGTGCTATCAGC |
| dHip14 5´ UTR R | tctggtacatCGCTGGCTTCTCTCGGTC |
| dHip14-BioID-HA F | gaagccagcgATGTACCAGAGTGCCTGC |
| dHip14-BioID-HA R | aggtcttatcCTATGCGTAATCCGGTACATC |
| dHip143´ UTR F | ttacgcatagGATAAGACCTATCGGGATGC |
| dHip143´ UTR R | ggttccttcacaaagatcctctagaAACGCACATTGTTACACATAC |
|  |  |
| dSnap24P120A |  |
| SNAP24 P120A F | gtggccagccaggcgcaaagggtca |
| SNAP24 P120A R | tgaccctttgcgcctggctggccac |
| dSnap25 P125A |  |
| SNAP25 P125A F | ggaaaagttgtaaataatcaggcacagagagtgatggatgata |
| SNAP25 P125A R | tatcatccatcactctctgtgcctgattatttacaacttttcc |
|  |  |
