# Supplementary material for: The S-palmitoylome and DHHC-PAT interactome of Drosophila melanogaster S2R+ cells indicate a high degree of conservation to mammalian palmitoylomes
Source: PLoS One. 2022 Aug 12;17(8):e0261543. doi: 10.1371/journal.pone.0261543 (PMC9374236; doi:10.1371/journal.pone.0261543)
Supplement: S2 Table — Putative palmitoylated proteins identified both by acyl-RAC in S2R+ cells (this study) and by ABE in Drosophila larvae (Strassburger et al 2019). (DOCX) [file pone.0261543.s009.docx]

**S2 Table. Best-supported palmitoylated proteins in Drosophila.** Putative palmitoylated proteins identified both by acyl-RAC in S2R+ cells (this study) and by ABE in Drosophila larvae (Strassburger *et al*).

| Flybase ID | UniProt ID | Gene symbol | Description |
| --- | --- | --- | --- |
| FBgn0037339 | Q8STF7_DROME | Pi4KIIalpha | Phosphatidylinositol 4-kinase alpha type II |
| FBgn0038407 | Q961R9_DROME | CT19169 | Transmembrane transporter protein |
| FBgn0034057 | Q7K3T4_DROME | CG8314 | Palmitoyltransferase |
| FBgn0036816 | INDY1_DROME | Indy | Protein I'm not dead yet |
| FBgn0260857 | Q9VVX6_DROME | Bet1 | Blocked early in transport 1 |
| FBgn0260859 | Q9VSY8_DROME | Bet3 | Trafficking protein particle complex subunit |
| FBgn0033199 | Q7K4J7_DROME | CG17985 | LD36653p |
| FBgn0039291 | Q9VBW0_DROME | CG13663 | SAYSvFN domain-containing protein |
| FBgn0052850 | Q8SX35_DROME | RNF-11 | Ubiquitin ligase |
| FBgn0039427 | Q9VBF0_DROME | CG5447 | Uncharacterized protein |
| FBgn0037383 | Q9VNI9_DROME | Sec20 | Vesicle transport protein |
| FBgn0001104 | GNAI_DROME | Galphai | G protein alpha i subunit |
| FBgn0250823 | Q9VEX2_DROME | gish | Protein Gilgamesh |
| FBgn0028506 | Q9VJK9_DROME | BG:DS09218.3 | Uncharacterized transmembrane protein |
| FBgn0036932 | Q9VW73_DROME | p18 | Lipid raft adaptor protein p18 |
| FBgn0012051 | CANA_DROME | CalpA | Calpain-A |
| FBgn0034399 | Q7JYX3_DROME | CG15083 | Uncharacterized transmembrane protein |
| FBgn0264078 | FLOT2_DROME | Flo2 | Flotillin-2 |
| FBgn0033127 | Q7K010_DROME | Tsp42Ef | Tetraspanin |
| FBgn0035165 | Q9W0M4_DROME | CG13887 | Uncharacterized protein |
| FBgn0261722 | FLOWR_DROME | fwe | Calcium channel flower |
| FBgn0001122 | GNAO_DROME | Galphao | G protein alpha o subunit |
| FBgn0001123 | GNAS_DROME | Galphas | G protein alpha s subunit |
| FBgn0259170 | MA1A1_DROME | alpha-Man-Ia | Mannosyl-oligosaccharide alpha-1,2-mannosidase IA |
| FBgn0015286 | RALA_DROME | Rala | Ras-related protein Ral-a |
| FBgn0033259 | Q6NP91_DROME | CG11210 | Uncharacterized protein |
| FBgn0034914 | Q9W1I7_DROME | CG5554 | Thioredoxin |
| FBgn0283666 | O96692_DROME | Rap2l | Ras-associated protein 2-like |
| FBgn0035965 | USE1_DROME | Use1 | Vesicle transport protein USE1 |
| FBgn0262515 | Q7JR49_DROME | VhaAC45 | V-type proton ATPase subunit |
| FBgn0028394 | Q6NL77_DROME | CG17834 | Uncharacterized protein |
| FBgn0004179 | DNJC5_DROME | Csp | Cysteine string protein. DnaJ homolog subfamily C member 5 homolog |
| FBgn0032378 | Q9VKF0_DROME | CycY | Cyclin Y |
| FBgn0039665 | Q7JZY1_DROME | CG2310 | Uncharacterized transmembrane protein |
| FBgn0027453 | Q9VKN8_DROME | Dnz1 | Palmitoyltransferase |
| FBgn0052536 | Q9VWJ3_DROME | CG8020 | Uncharacterized protein |
| FBgn0031681 | GALT5_DROME | pgant5 | Polypeptide N-acetylgalactosaminyltransferase 5 |
| FBgn0030930 | GALT7_DROME | Pgant7 | N-acetylgalactosaminyltransferase 7 |
| FBgn0033919 | Q961R5_DROME | CG8547 | Uncharacterized protein |
| FBgn0033913 | Q7K1L4_DROME | CG8468 | Uncharacterized transmembrane protein |
| FBgn0035519 | Q9VZF1_DROME | CG1309 | Uncharacterized transmembrane protein |
| FBgn0029506 | Q8SY17_DROME | Tsp42Ee | Tetraspanin |
| FBgn0029507 | Q7JWV7_DROME | Tsp42Ed | Tetraspanin |
| FBgn0026076 | Q9VXN1_DROME | UBL3 | HCG-1 protein |
| FBgn0032949 | Q9V9S0_DROME | Lamp1 | Uncharacterized transmembrane protein |
| FBgn0035523 | CTLH1_DROME | CG1311 | Choline transporter-like 1 |
| FBgn0052066 | Q7K1H0_DROME | CG6491 | Uncharacterized protein |
| FBgn0034494 | Q8SWV5_DROME | CG10444 | Uncharacterized transmembrane protein |
| FBgn0266720 | Q9VH76_DROME | Snap24 | Synaptosomal-associated protein |
| FBgn0010549 | L259_DROME | l(2)03659 | Probable multidrug resistance-associated protein lethal(2)03659 |
| FBgn0024754 | FLOT1_DROME | Flo1 | Flotillin-1 |
| FBgn0259824 | HIP14_DROME | Hip14 | Palmitoyltransferase Hip14 |
| FBgn0037465 | Q9VI53_DROME | CG1105 | Arrestin domain-containing protein |
| FBgn0004435 | GNAQ_DROME | Galphaq | G protein alpha q subunit |
| FBgn0086784 | EFR3_DROME | stmA | Protein EFR3 homolog cmp44E |
| FBgn0030993 | Q9VWL0_DROME | Mec2 | PHB domain-containing protein |
| FBgn0032916 | Q9VIG0_DROME | CG9257 | Malectin domain-containing protein |
| FBgn0053096 | Q9VBX8_DROME | CG11935 | Uncharacterized protein |
| FBgn0025820 | O96067_DROME | JTBR | DJTB-like protein |
| FBgn0052056 | Q8IQD7_DROME | scramb1 | Phospholipid scramblase |
| FBgn0030744 | Q9VXH0_DROME | CG9992 | Uncharacterized protein |
