# Supplementary material for: The S-palmitoylome and DHHC-PAT interactome of Drosophila melanogaster S2R+ cells indicate a high degree of conservation to mammalian palmitoylomes
Source: PLoS One. 2022 Aug 12;17(8):e0261543. doi: 10.1371/journal.pone.0261543 (PMC9374236; doi:10.1371/journal.pone.0261543)
Supplement: S3 Table — Fraction of Drosophila proteins with mammalian orthologs, and known palmitoylated mammalian orthologs under two confidence levels. (DOCX) [file pone.0261543.s010.docx]

## **S3 Table. Assessment of the conservation of Drosophila palmitoylated proteins in mammals.** Fraction of *Drosophila* proteins with mammalian ortholog, and known palmitoylated mammalian ortholog under two confidence levels.

|  |  |  | *Drosophila* proteins with mammalian ortholog | | | | | | | |
| --- | --- | --- | --- | --- | --- | --- | --- | --- | --- | --- |
| Dataset | Cut-offs | Total  proteins | with orthologs | % of total | palmitoylated mamm. orthologs* | % of total | % of ortho. | palmitoylated mamm. ortho. HC** | % of total | % of ortho. |
| Complete proteome | - | 13788 | 7287 | 52.9 | 2351 | 17.1 | 32.3 | 1234 | 8.9 | 16.9 |
| Acyl-RAC: not palmitoylated | No Cys or  FDR>=0.1 or FC<2 | 990 | 878 | 88.7 | 620 | 62.6 | 70.6 | 431 | 43.5 | 49.1 |
| Acyl-RAC: palmitoylated | with Cys  FDR <0.1  20 > FC >= 2 | 147 | 123 | 83.7 | 83 | 56.5 | 67.5 | 66 | 44.9 | 53.7 |
| Acyl-RAC: palmitoylated (HC) | with Cys  FDR < 0.1  FC>= 20 | 51 | 43 | 84.3 | 32 | 62.7 | 74.4 | 27 | 52.9 | 62.8 |

mamm. ortho. = mammalian orthologs identified for Drosophila proteins in the respective dataset;

% of total refers to the total dataset in a row (first data-column); % of ortho. refers to the number of proteins with mammalian orthologs (second data-column).

* palmitoylated proteins: SwissPalm database, all reported mammalian entries.

** increased confidence: SwissPalm database, mammalian entries filtered for entries reported at least in 2 studies.
