# Supplementary material for: The S-palmitoylome and DHHC-PAT interactome of Drosophila melanogaster S2R+ cells indicate a high degree of conservation to mammalian palmitoylomes
Source: PLoS One. 2022 Aug 12;17(8):e0261543. doi: 10.1371/journal.pone.0261543 (PMC9374236; doi:10.1371/journal.pone.0261543)
Supplement: S4 Table — Overrepresented GO terms associated to the 198 S-palmitoylated proteins were determined with Fisher’s exact tests, using the whole D. melanogaster’s proteome as reference. FDR correction was applied to Fisher’s test P-values to adjust for multiple testing. GO terms were considered significantly enriched if FDR adjusted P-value < 0.05. GO terms are separated in the three main ontologies (cellular component, biological process and molecular function) and sorted by their FDR adjusted P-value. (DOCX) [file pone.0261543.s011.docx]

## **S4 Table. Complete GO enrichment analysis results of the *D. melanogaster* S2R+ cell palmitoylome proteins.** Overrepresented GO terms associated to the 198 S-palmitoylated proteins were determined with Fisher’s exact tests, using the whole *D. melanogaster*’s proteome as reference. FDR correction was applied to Fisher’s test P-values to adjust for multiple testing. GO terms were considered significantly enriched if FDR adjusted P-value < 0.05. GO terms are separated in the three main ontologies (cellular component, biological process and molecular function) and sorted by their FDR adjusted P-value.

| **Ontology** | **GO Accession** | **GO Name** | **In Reference D.Mel Proteome** | **Found in Dataset** | **Expected** | **Over/Under** | **Fold Enrichment** | **Raw P-value** | **FDR (Adj. P-value)** |
| --- | --- | --- | --- | --- | --- | --- | --- | --- | --- |
| **Cellular Component** |  |  |  |  |  |  |  |  |  |
|  | GO:0016020 | membrane | 2760 | 121 | 39.65 | + | 3.05 | 9.32E-36 | 1.25E-32 |
|  | GO:0012505 | endomembrane system | 1138 | 59 | 16.35 | + | 3.61 | 3.50E-18 | 2.35E-15 |
|  | GO:0031224 | intrinsic component of membrane | 1466 | 66 | 21.06 | + | 3.13 | 1.94E-17 | 8.68E-15 |
|  | GO:0016021 | integral component of membrane | 1445 | 64 | 20.76 | + | 3.08 | 1.58E-16 | 5.31E-14 |
|  | GO:0005886 | plasma membrane | 1300 | 59 | 18.68 | + | 3.16 | 1.31E-15 | 3.51E-13 |
|  | GO:0005794 | Golgi apparatus | 368 | 30 | 5.29 | + | 5.67 | 5.45E-14 | 1.22E-11 |
|  | GO:0071944 | cell periphery | 1446 | 59 | 20.78 | + | 2.84 | 1.25E-13 | 2.39E-11 |
|  | GO:0098588 | bounding membrane of organelle | 338 | 23 | 4.86 | + | 4.74 | 1.66E-09 | 2.78E-07 |
|  | GO:0031410 | cytoplasmic vesicle | 404 | 24 | 5.8 | + | 4.13 | 8.91E-09 | 1.19E-06 |
|  | GO:0110165 | cellular anatomical entity | 8747 | 163 | 125.67 | + | 1.3 | 9.95E-09 | 1.21E-06 |
|  | GO:0097708 | intracellular vesicle | 404 | 24 | 5.8 | + | 4.13 | 8.91E-09 | 1.33E-06 |
|  | GO:0031982 | vesicle | 441 | 24 | 6.34 | + | 3.79 | 4.37E-08 | 4.88E-06 |
|  | GO:0098562 | cytoplasmic side of membrane | 52 | 9 | 0.75 | + | 12.05 | 1.76E-07 | 1.81E-05 |
|  | GO:0005768 | endosome | 226 | 16 | 3.25 | + | 4.93 | 3.46E-07 | 3.31E-05 |
|  | GO:0098791 | Golgi subcompartment | 104 | 11 | 1.49 | + | 7.36 | 6.87E-07 | 6.15E-05 |
|  | GO:0009898 | cytoplasmic side of plasma membrane | 46 | 8 | 0.66 | + | 12.1 | 8.37E-07 | 7.02E-05 |
|  | GO:0000139 | Golgi membrane | 87 | 10 | 1.25 | + | 8 | 1.12E-06 | 8.82E-05 |
|  | GO:0098552 | side of membrane | 68 | 9 | 0.98 | + | 9.21 | 1.34E-06 | 1.00E-04 |
|  | GO:0031984 | organelle subcompartment | 113 | 11 | 1.62 | + | 6.78 | 1.46E-06 | 1.03E-04 |
|  | GO:0005770 | late endosome | 72 | 9 | 1.03 | + | 8.7 | 2.07E-06 | 1.39E-04 |
|  | GO:0005783 | endoplasmic reticulum | 335 | 18 | 4.81 | + | 3.74 | 2.79E-06 | 1.78E-04 |
|  | GO:1905360 | GTPase complex | 13 | 5 | 0.19 | + | 26.77 | 4.00E-06 | 2.33E-04 |
|  | GO:0005834 | heterotrimeric G-protein complex | 13 | 5 | 0.19 | + | 26.77 | 4.00E-06 | 2.44E-04 |
|  | GO:0031090 | organelle membrane | 801 | 29 | 11.51 | + | 2.52 | 5.84E-06 | 3.26E-04 |
|  | GO:0005795 | Golgi stack | 64 | 8 | 0.92 | + | 8.7 | 7.75E-06 | 4.16E-04 |
|  | GO:0098805 | whole membrane | 259 | 14 | 3.72 | + | 3.76 | 3.48E-05 | 1.79E-03 |
|  | GO:0098797 | plasma membrane protein complex | 165 | 11 | 2.37 | + | 4.64 | 4.17E-05 | 2.00E-03 |
|  | GO:0031234 | extrinsic component of cytoplasmic side of plasma membrane | 23 | 5 | 0.33 | + | 15.13 | 4.09E-05 | 2.03E-03 |
|  | GO:0010008 | endosome membrane | 63 | 7 | 0.91 | + | 7.73 | 5.80E-05 | 2.68E-03 |
|  | GO:0031226 | intrinsic component of plasma membrane | 472 | 19 | 6.78 | + | 2.8 | 7.12E-05 | 3.18E-03 |
|  | GO:0045121 | membrane raft | 13 | 4 | 0.19 | + | 21.42 | 8.04E-05 | 3.27E-03 |
|  | GO:0098857 | membrane microdomain | 13 | 4 | 0.19 | + | 21.42 | 8.04E-05 | 3.37E-03 |
|  | GO:0031902 | late endosome membrane | 27 | 5 | 0.39 | + | 12.89 | 8.00E-05 | 3.46E-03 |
|  | GO:0098589 | membrane region | 15 | 4 | 0.22 | + | 18.56 | 1.28E-04 | 5.05E-03 |
|  | GO:0005887 | integral component of plasma membrane | 459 | 18 | 6.59 | + | 2.73 | 1.52E-04 | 5.83E-03 |
|  | GO:0005575 | cellular component | 10729 | 175 | 154.15 | + | 1.14 | 1.88E-04 | 7.01E-03 |
|  | GO:0098796 | membrane protein complex | 521 | 19 | 7.49 | + | 2.54 | 2.44E-04 | 8.39E-03 |
|  | GO:0031228 | intrinsic component of Golgi membrane | 35 | 5 | 0.5 | + | 9.94 | 2.38E-04 | 8.41E-03 |
|  | GO:0005634 | nucleus | 2712 | 20 | 38.96 | - | 0.51 | 3.99E-04 | 1.27E-02 |
|  | GO:0005765 | lysosomal membrane | 21 | 4 | 0.3 | + | 13.26 | 3.91E-04 | 1.28E-02 |
|  | GO:0098852 | lytic vacuole membrane | 21 | 4 | 0.3 | + | 13.26 | 3.91E-04 | 1.31E-02 |
|  | GO:0019897 | extrinsic component of plasma membrane | 41 | 5 | 0.59 | + | 8.49 | 4.64E-04 | 1.45E-02 |
|  | GO:0005764 | lysosome | 92 | 7 | 1.32 | + | 5.3 | 5.10E-04 | 1.55E-02 |
|  | GO:0000323 | lytic vacuole | 93 | 7 | 1.34 | + | 5.24 | 5.42E-04 | 1.61E-02 |
|  | GO:0005774 | vacuolar membrane | 67 | 6 | 0.96 | + | 6.23 | 5.79E-04 | 1.69E-02 |
|  | GO:0022627 | cytosolic small ribosomal subunit | 44 | 5 | 0.63 | + | 7.91 | 6.23E-04 | 1.78E-02 |
|  | GO:0000137 | Golgi cis cisterna | 10 | 3 | 0.14 | + | 20.88 | 7.21E-04 | 2.01E-02 |
|  | GO:0016600 | flotillin complex | 2 | 2 | 0.03 | + | 69.6 | 1.18E-03 | 3.22E-02 |
|  | GO:0031201 | SNARE complex | 30 | 4 | 0.43 | + | 9.28 | 1.30E-03 | 3.48E-02 |
|  | GO:0031300 | intrinsic component of organelle membrane | 181 | 9 | 2.6 | + | 3.46 | 1.55E-03 | 4.08E-02 |
|  | GO:0030173 | integral component of Golgi membrane | 33 | 4 | 0.47 | + | 8.44 | 1.79E-03 | 4.61E-02 |
|  | GO:0005901 | caveola | 3 | 2 | 0.04 | + | 46.4 | 1.94E-03 | 4.82E-02 |
|  | GO:0036019 | endolysosome | 3 | 2 | 0.04 | + | 46.4 | 1.94E-03 | 4.91E-02 |
| **Biological Process** |  |  |  |  |  |  |  |  |  |
|  | GO:0051234 | establishment of localization | 1716 | 75 | 24.65 | + | 3.04 | 2.17E-19 | 1.70E-15 |
|  | GO:0051179 | localization | 2174 | 84 | 31.24 | + | 2.69 | 9.05E-19 | 3.55E-15 |
|  | GO:0006810 | transport | 1659 | 71 | 23.84 | + | 2.98 | 9.66E-18 | 2.53E-14 |
|  | GO:0071702 | organic substance transport | 733 | 33 | 10.53 | + | 3.13 | 9.10E-09 | 1.79E-05 |
|  | GO:0018345 | protein palmitoylation | 25 | 8 | 0.36 | + | 22.27 | 1.44E-08 | 2.26E-05 |
|  | GO:0016192 | vesicle-mediated transport | 468 | 25 | 6.72 | + | 3.72 | 3.15E-08 | 4.12E-05 |
|  | GO:0042157 | lipoprotein metabolic process | 72 | 10 | 1.03 | + | 9.67 | 2.29E-07 | 2.56E-04 |
|  | GO:0055085 | transmembrane transport | 611 | 27 | 8.78 | + | 3.08 | 3.41E-07 | 3.35E-04 |
|  | GO:0018230 | peptidyl-L-cysteine S-palmitoylation | 20 | 6 | 0.29 | + | 20.88 | 1.36E-06 | 7.63E-04 |
|  | GO:0042158 | lipoprotein biosynthetic process | 67 | 9 | 0.96 | + | 9.35 | 1.20E-06 | 7.86E-04 |
|  | GO:0018231 | peptidyl-S-diacylglycerol-L-cysteine biosynthetic process from peptidyl-cysteine | 20 | 6 | 0.29 | + | 20.88 | 1.36E-06 | 8.22E-04 |
|  | GO:0006497 | protein lipidation | 67 | 9 | 0.96 | + | 9.35 | 1.20E-06 | 8.58E-04 |
|  | GO:0033036 | macromolecule localization | 866 | 32 | 12.44 | + | 2.57 | 1.17E-06 | 9.22E-04 |
|  | GO:0071705 | nitrogen compound transport | 612 | 26 | 8.79 | + | 2.96 | 1.17E-06 | 1.02E-03 |
|  | GO:0018198 | peptidyl-cysteine modification | 23 | 6 | 0.33 | + | 18.16 | 2.71E-06 | 1.42E-03 |
|  | GO:0033619 | membrane protein proteolysis | 12 | 5 | 0.17 | + | 29 | 2.92E-06 | 1.43E-03 |
|  | GO:0006811 | ion transport | 498 | 22 | 7.16 | + | 3.07 | 4.59E-06 | 2.12E-03 |
|  | GO:0006612 | protein targeting to membrane | 42 | 7 | 0.6 | + | 11.6 | 5.34E-06 | 2.33E-03 |
|  | GO:0015711 | organic anion transport | 139 | 11 | 2 | + | 5.51 | 9.35E-06 | 3.86E-03 |
|  | GO:0048193 | Golgi vesicle transport | 141 | 11 | 2.03 | + | 5.43 | 1.06E-05 | 4.16E-03 |
|  | GO:0045184 | establishment of protein localization | 492 | 21 | 7.07 | + | 2.97 | 1.26E-05 | 4.73E-03 |
|  | GO:0046483 | heterocycle metabolic process | 1427 | 4 | 20.5 | - | 0.2 | 1.42E-05 | 5.06E-03 |
|  | GO:0006820 | anion transport | 208 | 13 | 2.99 | + | 4.35 | 1.59E-05 | 5.44E-03 |
|  | GO:0008104 | protein localization | 715 | 26 | 10.27 | + | 2.53 | 1.74E-05 | 5.45E-03 |
|  | GO:0023052 | signaling | 1170 | 36 | 16.81 | + | 2.14 | 1.67E-05 | 5.46E-03 |
|  | GO:0090150 | establishment of protein localization to membrane | 75 | 8 | 1.08 | + | 7.42 | 2.24E-05 | 6.75E-03 |
|  | GO:0006509 | membrane protein ectodomain proteolysis | 9 | 4 | 0.13 | + | 30.93 | 2.53E-05 | 7.35E-03 |
|  | GO:0046907 | intracellular transport | 646 | 24 | 9.28 | + | 2.59 | 2.70E-05 | 7.56E-03 |
|  | GO:0006139 | nucleobase-containing compound metabolic process | 1347 | 4 | 19.35 | - | 0.21 | 2.86E-05 | 7.75E-03 |
|  | GO:0006725 | cellular aromatic compound metabolic process | 1471 | 5 | 21.13 | - | 0.24 | 3.35E-05 | 8.77E-03 |
|  | GO:0098657 | import into cell | 162 | 11 | 2.33 | + | 4.73 | 3.56E-05 | 9.02E-03 |
|  | GO:0051641 | cellular localization | 934 | 30 | 13.42 | + | 2.24 | 4.72E-05 | 1.16E-02 |
|  | GO:0007154 | cell communication | 1237 | 36 | 17.77 | + | 2.03 | 5.18E-05 | 1.23E-02 |
|  | GO:1901360 | organic cyclic compound metabolic process | 1526 | 6 | 21.92 | - | 0.27 | 7.54E-05 | 1.74E-02 |
|  | GO:0007267 | cell-cell signaling | 318 | 15 | 4.57 | + | 3.28 | 8.06E-05 | 1.81E-02 |
|  | GO:0090304 | nucleic acid metabolic process | 1086 | 3 | 15.6 | - | 0.19 | 1.51E-04 | 3.20E-02 |
|  | GO:0031293 | membrane protein intracellular domain proteolysis | 5 | 3 | 0.07 | + | 41.76 | 1.49E-04 | 3.24E-02 |
|  | GO:0072657 | protein localization to membrane | 135 | 9 | 1.94 | + | 4.64 | 2.10E-04 | 4.33E-02 |
|  | GO:0015031 | protein transport | 475 | 18 | 6.82 | + | 2.64 | 2.28E-04 | 4.60E-02 |
|  | GO:0015833 | peptide transport | 479 | 18 | 6.88 | + | 2.62 | 2.52E-04 | 4.94E-02 |
| **Molecular Function** |  |  |  |  |  |  |  |  |  |
|  | GO:0005215 | transporter activity | 791 | 34 | 11.36 | + | 2.99 | 1.59E-08 | 4.52E-05 |
|  | GO:0016409 | palmitoyltransferase activity | 31 | 8 | 0.45 | + | 17.96 | 5.94E-08 | 8.45E-05 |
|  | GO:0019706 | protein-cysteine S-palmitoyltransferase activity | 23 | 7 | 0.33 | + | 21.18 | 1.59E-07 | 1.13E-04 |
|  | GO:0019707 | protein-cysteine S-acyltransferase activity | 23 | 7 | 0.33 | + | 21.18 | 1.59E-07 | 1.51E-04 |
|  | GO:0016417 | S-acyltransferase activity | 26 | 7 | 0.37 | + | 18.74 | 3.22E-07 | 1.83E-04 |
|  | GO:0031683 | G-protein beta/gamma-subunit complex binding | 8 | 5 | 0.11 | + | 43.5 | 6.36E-07 | 3.02E-04 |
|  | GO:0022857 | transmembrane transporter activity | 733 | 28 | 10.53 | + | 2.66 | 3.24E-06 | 1.32E-03 |
|  | GO:0008514 | organic anion transmembrane transporter activity | 110 | 9 | 1.58 | + | 5.69 | 4.83E-05 | 1.72E-02 |
|  | GO:0008509 | anion transmembrane transporter activity | 176 | 11 | 2.53 | + | 4.35 | 7.24E-05 | 2.29E-02 |
|  | GO:0015075 | ion transmembrane transporter activity | 538 | 20 | 7.73 | + | 2.59 | 1.30E-04 | 3.71E-02 |
