# Supplementary material for: The S-palmitoylome and DHHC-PAT interactome of Drosophila melanogaster S2R+ cells indicate a high degree of conservation to mammalian palmitoylomes
Source: PLoS One. 2022 Aug 12;17(8):e0261543. doi: 10.1371/journal.pone.0261543 (PMC9374236; doi:10.1371/journal.pone.0261543)

# S1 Appendix. Optimization of experimental conditions for the use of BioID in Drosophila S2R+ cells

To date, BioID has not been successfully used in Drosophila. Originally, BioID was developed for organisms adapted to 37 °C. Drosophila does not tolerate temperatures above 32 °C [1] and Drosophila S2R+ cells also died quickly after a shift in temperature from 25 °C to 37 °C (Appendix Fig 1). Hence, neither flies nor Drosophila cells can be cultured at the temperature that BioID or BioID2 are usually used at (37 °C). Therefore, we carried out pilot experiments to determine the optimal conditions for the use of BioID and BioID2 in S2R+ cells. myc-BioID was successfully expressed using the pMt-Gal4/UASt system and resulted in low protein biotinylation activity in medium that was not supplemented with biotin (Appendix Fig 2A). BioID activity was markedly increased in cells grown in medium supplemented with 50 μM biotin, while 300 μM biotin did not further increase biotinylated protein abundance (Appendix Fig 2A). Cells grown at 30 °C showed slightly increased myc-BioID expression and 2-fold higher biotinylation activity compared to cells grown at 25 °C (Appendix Figs 2A and 3). These conditions were then used to compare the activities of BioID and BioID2. Cells harvested 24, 48 and 72 h after induction of expression showed comparable levels of expression of the biotin ligases and protein biotinylation increased with longer incubation time (Appendix Fig 2B). Relative quantification of biotinylation signals showed that in the presence of 50 μM biotin, BioID had higher activity compared to BioID2 (Appendix Fig 2C). This is in contrast to the superior performance of BioID2 in mammalian systems [2]. Therefore, we proceeded to use BioID under the optimized conditions determined above (i.e. 30 °C, 50 μM biotin and incubation times of 48 h) in all subsequent experiments with the S2R+ cell line. Moreover, BioID is also feasible in larvae and adult flies (Appendix Figs 4 and 5). When using strong drivers or ubiquitous expression, myc-BioID is efficiently expressed, allowing the use of whole-animal lysates. However, in low-abundant cell types BioID did not yield enough biotinylated proteins to be efficiently detected in whole-animal homogenates. In that case, enrichment of body parts abundant in the target cell type (e.g. adult heads for neurons Appendix Fig 4D) or use of TurboID [3], a recently bioengineered high-efficiency variant of BioID, may be recommended.

## **Appendix References**

[1] M. Ashburner, K.G. Golic, R.S. Hawley, Drosophila : a laboratory handbook, (2005) 1409.

[2] D.I. Kim, S.C. Jensen, K.A. Noble, B. Kc, K.H. Roux, K. Motamedchaboki, K.J. Roux, An improved smaller biotin ligase for BioID proximity labeling, Mol. Biol. Cell. 27 (2016) 1188–1196. https://doi.org/10.1091/MBC.E15-12-0844.

[3] T.C. Branon, J.A. Bosch, A.D. Sanchez, N.D. Udeshi, T. Svinkina, S.A. Carr, J.L. Feldman, N. Perrimon, A.Y. Ting, Efficient proximity labeling in living cells and organisms with TurboID, Nat. Biotechnol. 36 (2018) 880–898. https://doi.org/10.1038/NBT.4201.

## **Appendix Fig 1**. **S2R+ cells do not tolerate temperature shift from 25 to 37 °C**. Cells were cultured in Schneiders Drosophila medium with 10% FBS in the presence of antibiotics at 25 °C. 24h after seeding cells, the culture dishes were moved to an incubator set to 37 °C for the indicated times; cells were harvested by vigorous shaking as used during splitting of cultures and the fraction of dead cells was determined by the trypan blue exlusion method. The data represents one experiment, means +/- SD of three independent sample preparations and counts per time point.

## **Appendix Fig 2.** **Establishment of BioID in *Drosophila* S2R+ cells.** (A) Cells expressing soluble myc-BioID were incubated either at 25 or 30°C with medium containing indicated biotin concentration (0- 300 uM) and harvested 24 hours post induction. (B) Overexpression of myc-BioID and myc-BioID2 in S2R+ cells incubated at 30°C at different time points (24, 48 and 72 hours) post-induction. (A, B) Western blots representative of cells expressing myc-BioID or myc-BioID2 using the Gal4/UASt system and induced with CuSO_4_ 0.25 mM. myc-BioID was detected using an anti-myc primary antibody (upper panels) and biotin was detected using a streptavidin probe (lower panels) on the same blot membrane using two different fluorophores on a Li-Cor Odyssey. (C) Quantification of promiscuous biotinylation relative to BioID or BioID2 overexpression in S2R+ cells 48 hours after induction and grown in medium in absence (0 uM) or supplemented with 50 uM biotin. Biotinylation signals are normalized to myc-BioID or myc-BioID2 expression levels respectively. Results from three independent experiments are shown as means +/- standard deviations. Asterisks indicate statistically significant differences in relative biotinylation (*, P<0.05; **P<0.01) according to one-way ANOVA with Bonferroni’s multiple comparison test.

**
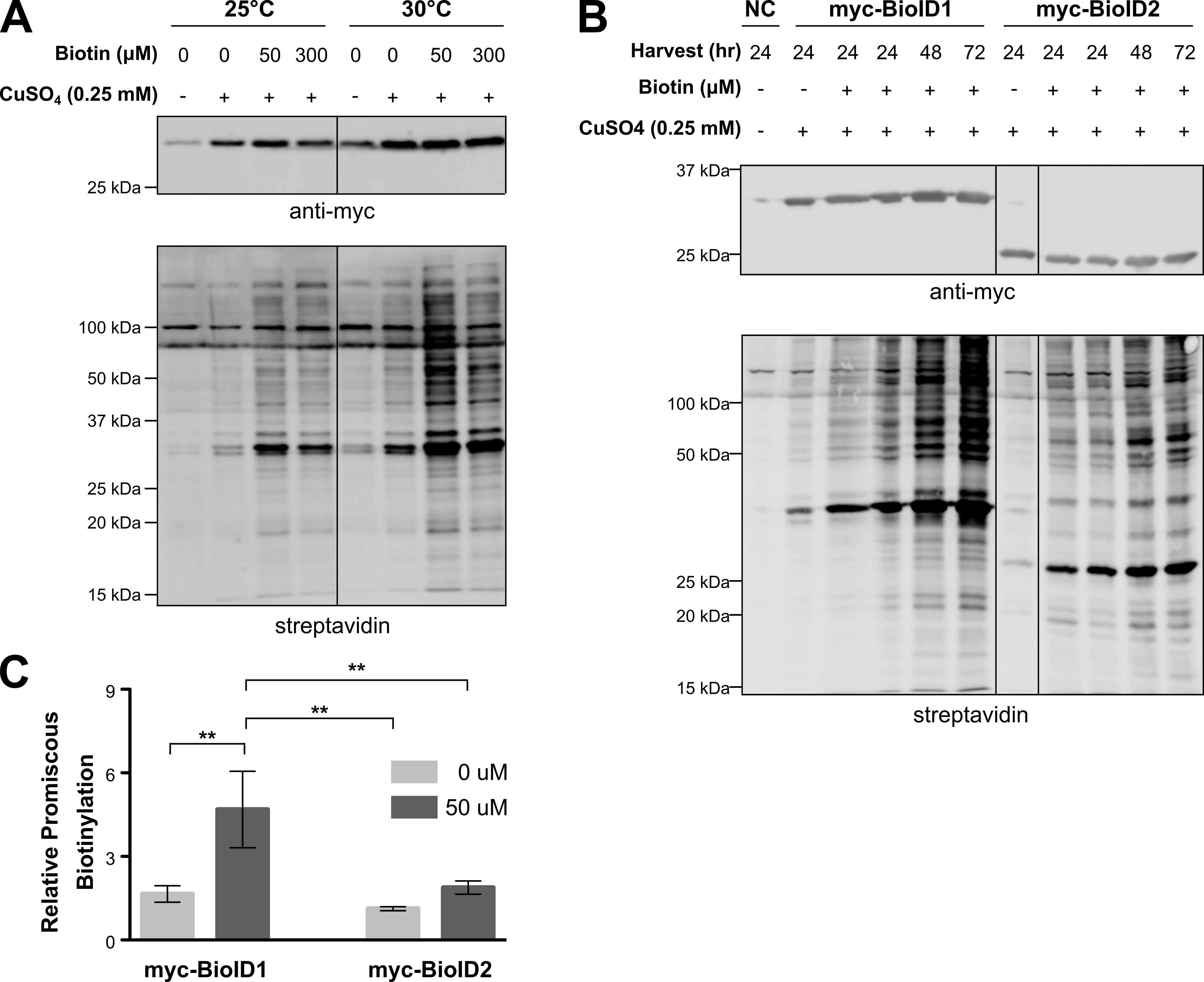
**

**Appendix Fig 3.** **Establishment of BioID in *Drosophila* S2R+ cells.** (A) Original blot from Appendix Fig 2A. (B) Original blot from Appendix Fig 2B. Boxed areas are relative to the cropped image in the main figure.


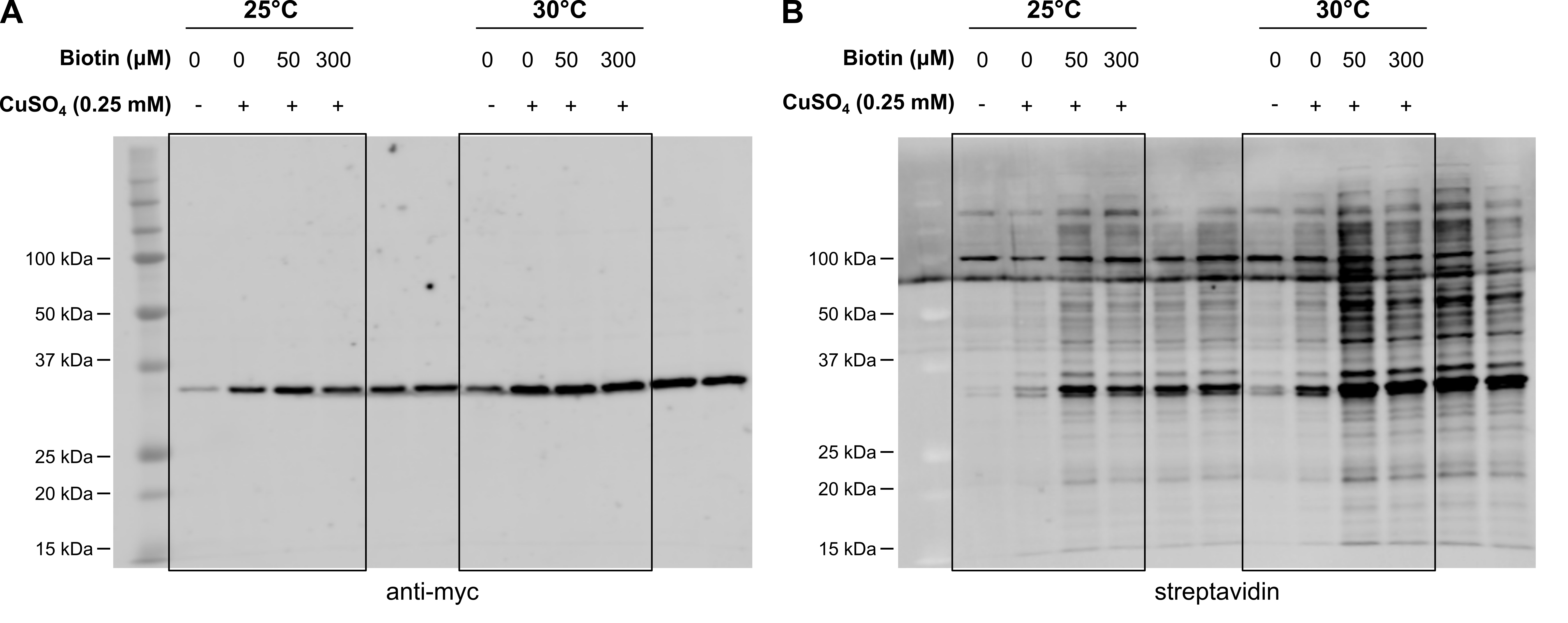


## **Appendix Fig 4**. **Quantification of biotinylation in S2R+ cells expressing mycBioID.** Cells were co-transfected with pMT-GAL4 and pUASt-mycBioID. Expression of mycBioID was induced by adding copper (0.25 mM final concentration) to the growth medium 24 h after transfection. Biotin at indicated concentrations (in uM) was added at the time point of induction and cells incubated at 30 °C were moved from 25 °C to 30 °C. 24 h after induction cells were harvested, lysed and used for western blot. Results of two independent transfections are shown. (A) upper panel: mycBioID detected by anti-myc antibody; lower panel: biotinylated proteins detected by streptavidin; (B) relative quantification of: mycBioID expression (upper panel, signals from anti-myc in A); autobiotinylation of myc-BioID (middle panel) and biotinylated proteins (lower panel) detected by streptavidin in A. To quantify autobiotinylated mycBioID the quantification boxes in ImageStudio were fitted around the signal corresponding to mycBioID at around 35 kDa. To relatively quantify other biotinylated proteins, the quantifcaiotn boxes were placed in the area from below the area with high backgound (approximately 60 kDa) until just above the mycBioID signal.


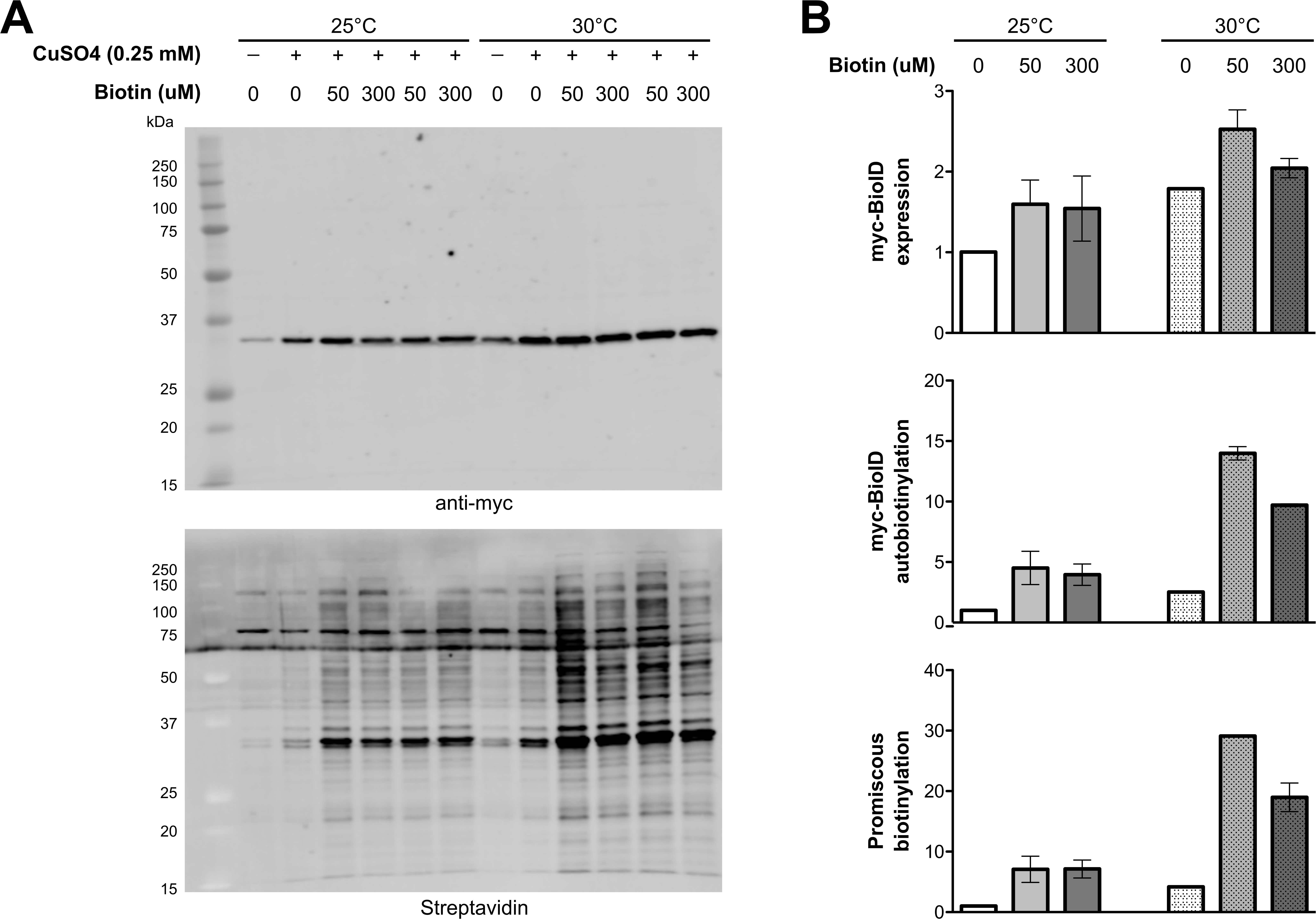


**Appendix Fig 5.** **Quantification of biotinylation in S2R+ cells expressing mycBioID.** Original blot from Appendix Fig 4A. (A) anti-myc (B) streptavidin. Boxed areas are relative to the cropped image in the main figure.

**
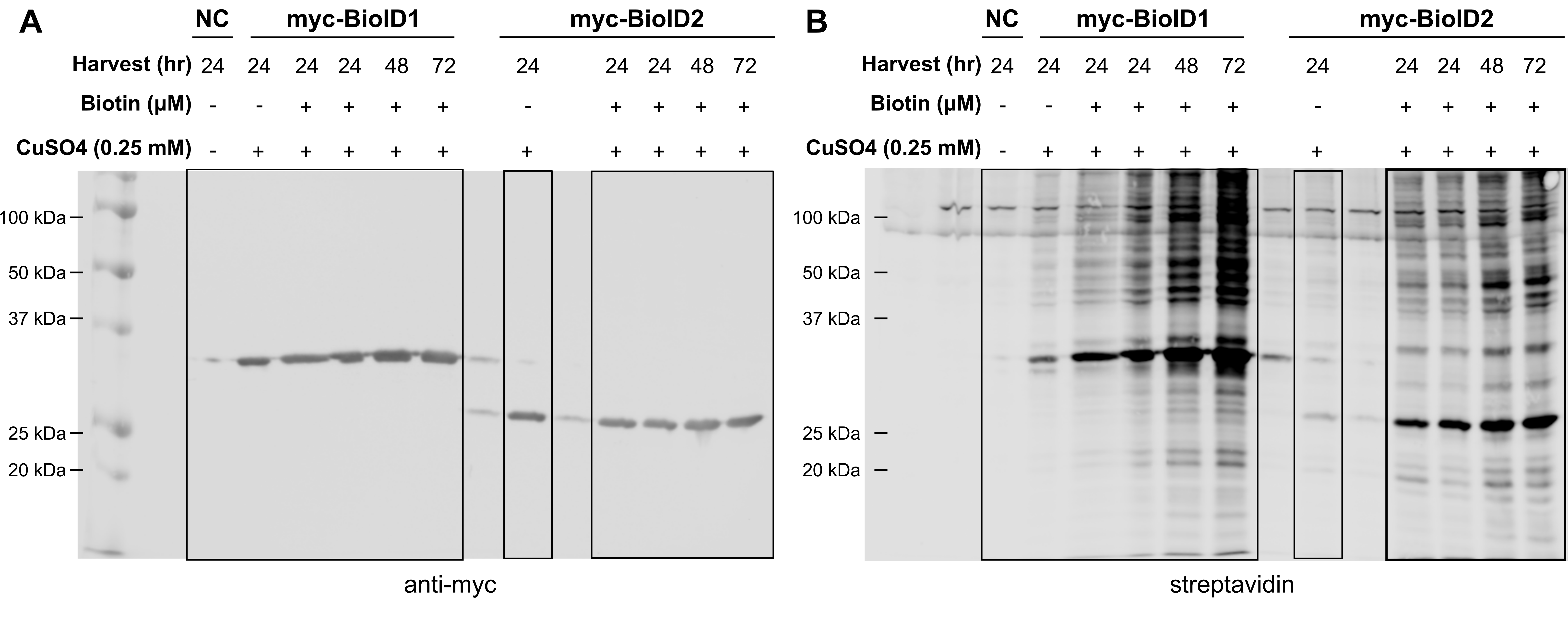
**

## **Appendix Fig 6**. **Establishment of BioID in *Drosophila*.** (A, B) Western blots representative of myc-BioID (A, B) or myc-BioID2 (B) expression and protein biotinylation of digging (DL), wandering (WL) larvae (A) or larvae grown at 30°C and placed on food with the indicated concentrations of biotin (0-400 mM). Male UAS-mycBioID and UAS-myc-BioID2 flies were crossed to female da-GAL4 flies. (C) Western blot representative of myc-BioID levels and biotinylation in larvae expressing myc-BioID at ubiquitous (u) or neuronal (n) levels, grown at different temperatures. (D) Western blot representative of myc-BioID expression in fly heads isolated from flies expressing at ubiquitous (u) or neuronal (n) levels and cultured at 30 °C. (C, D) Male UAS-mycBioID flies were crossed to female elav-GAL4 (n, neuronal) or da-GAL4 (u, ubiquitous) flies and grown on food supplemented with 400 uM biotin. Representative blots are shown. myc-BioID was detected using an anti-myc primary antibody (upper panels) and biotin was detected using a streptavidin probe (lower panels) on the same blot membrane using two different fluorophores on a Li-Cor Odyssey.


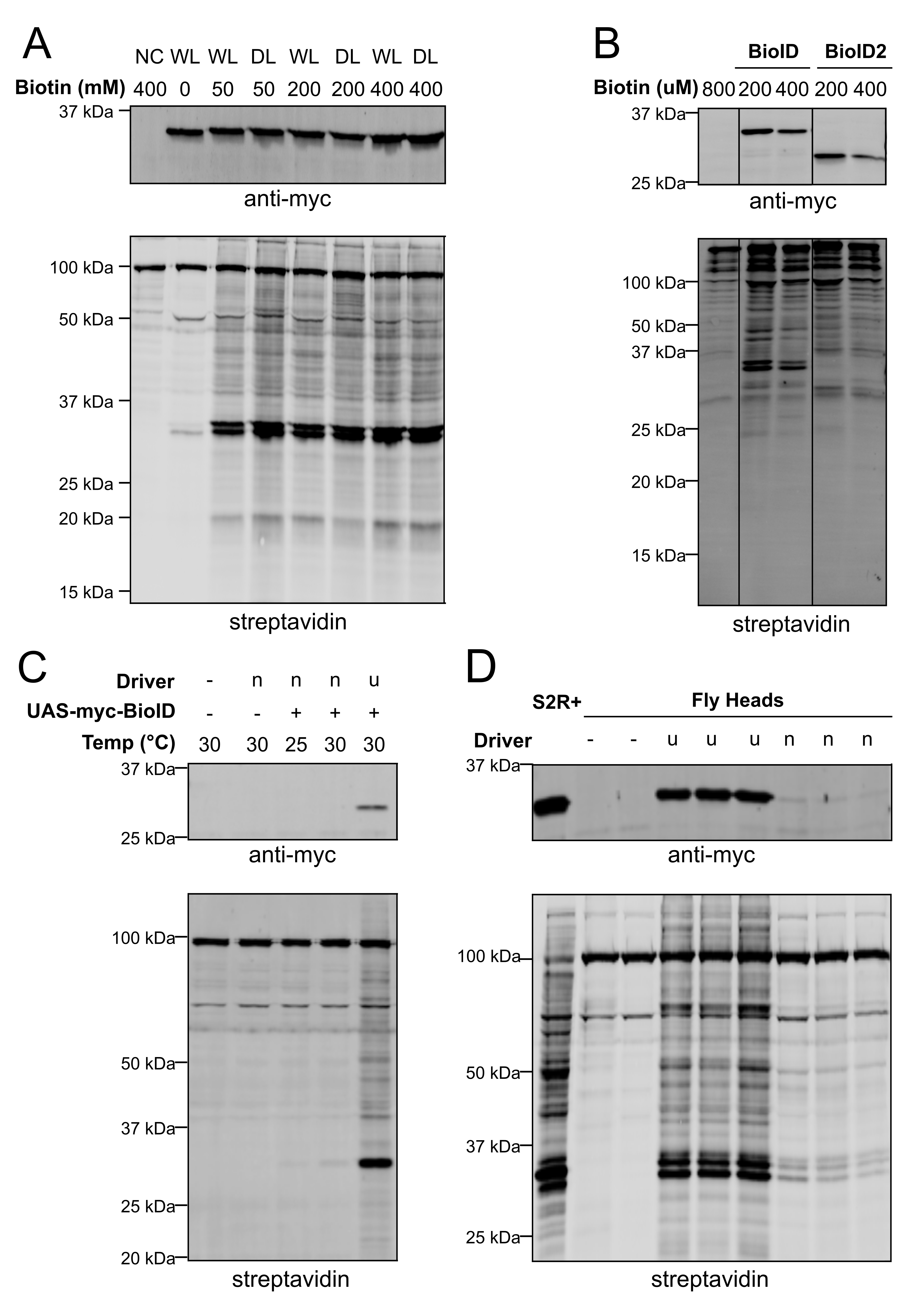


## **Appendix Fig 7**. **BioID works in all major body parts of the fly**. Protein biotinyltation can be observed in all body parts (thx = thorax; A = abdomen; H = head) of flies ubiquitously expressing myc-BioID (Actin-GAL4 +UASt-mycBioID). Whole fly lysate of USA-mycBioID flies without a driver were used as negative control (NC). All flies were reared on medium with 400 uM biotin supplementation and switched to 30 °C when first wandering third instar larvae were observed.


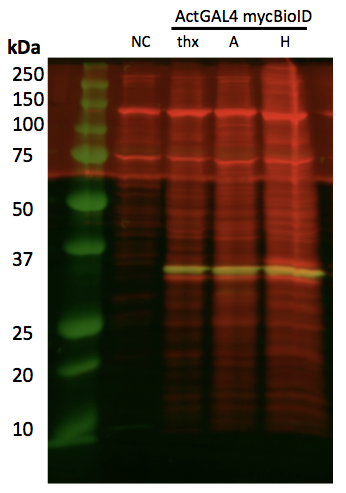

Supplement: S1 Appendix — Optimization of experimental conditions for the use of BioID in Drosophila S2R+ cells. (DOCX) [file pone.0261543.s012.docx]
